# Gender equity and menstrual justice: a psychosocial review on stigma and discrimination in access to reproductive health

**DOI:** 10.1186/s12978-025-02255-z

**Published:** 2026-01-07

**Authors:** Clara Selva, Mónica Natalia Butragueño, Carlota de Miquel

**Affiliations:** Estudis de Psicologia i Ciències de l’Educació (EPCE), Universitat Oberta de Catalunya (UOC), Barcelona, Spain

**Keywords:** Menstrual stigma, Discrimination, Menstruation, Menstrual cycle, Women, Menstrual poverty, Media, Legislation, Menstrual activism, Estigma menstrual, Discriminación, Menstruación, Ciclo menstrual, Mujer, Pobreza Menstrual, Medios de comunicación, Legislación, Activismo menstrual

## Abstract

**Background:**

The taboo and stigma surrounding menstruation remain deeply entrenched in diverse sociocultural contexts, generating significant inequalities in education, health, employment, and social participation. These barriers disproportionately affect individuals in vulnerable situations and are closely linked to what is known as menstrual poverty; defined as limited access to hygiene products, reliable information, and adequate water, sanitation, and hygiene (WASH) conditions. This review aims to critically analyse recent scientific literature on menstrual health and management, identifying key thematic areas, persistent inequalities, and proposed strategies across various social and geographical contexts.

**Methods:**

A narrative review was conducted of peer-reviewed literature published between 2019 and 2025 in international databases. From an initial pool of 905 records, 36 studies were selected based on pre-established inclusion and exclusion criteria. These studies employed diverse methodologies, including qualitative, quantitative, theoretical approaches, and literature reviews. The analysis was structured around six thematic axes: menstrual stigma, structural discrimination, menstrual poverty, media representations, labour legislation, and menstrual activism.

**Results:**

The reviewed studies demonstrate that menstrual stigma has a multidimensional impact, manifesting in social exclusion, medicalization of menstruating bodies, institutional neglect, and the persistence of discriminatory practices. Menstrual poverty, identified both in low-income and high-income settings, adversely affects the physical, emotional, and social well-being of menstruating individuals. Furthermore, the review highlights a lack of inclusive public policies, limited labour regulatory frameworks, and the reproduction of stigmatizing narratives in the media. Nevertheless, notable advances are emerging in the fields of activism, education, and legislative initiatives.

**Conclusions:**

The findings underscore the urgent need to develop intersectional public policies that approach menstrual health through a human rights, gender equity, and social justice lens. The review advocates for strengthened menstrual literacy, improved access to menstrual products and appropriate infrastructure, and the integration of menstrual health into educational, healthcare, and workplace systems as a pathway to ensure a dignified, healthy, and equitable menstrual experience.

## Resumen

**Antecedentes:**

El tabú y el estigma asociados a la menstruación siguen profundamente arraigados en múltiples contextos socioculturales, generando desigualdades significativas en ámbitos como la educación, la salud, el empleo y la participación social. Estas barreras afectan especialmente a personas en situación de vulnerabilidad y están vinculadas a lo que se ha conceptualizado como pobreza menstrual, entendida como la limitación en el acceso a productos de higiene, información fiable y condiciones adecuadas de agua, saneamiento e higiene. El objetivo de esta revisión es analizar críticamente la producción científica reciente sobre salud y gestión menstrual, identificando los principales ejes temáticos abordados, las desigualdades persistentes y las propuestas de actuación formuladas en distintos contextos sociales y geográficos.

**Métodos:**

Se realizó una revisión narrativa de la literatura científica publicada entre 2019 y 2025 en bases de datos internacionales. Tras aplicar criterios de inclusión y exclusión preestablecidos, de un total de 905 registros iniciales se seleccionaron 36 estudios con diferentes metodologías: cualitativos, cuantitativos, revisiones y estudios teóricos. El análisis se organizó en torno a seis ejes temáticos: estigma menstrual, discriminación estructural, pobreza menstrual, medios de comunicación, legislación laboral y activismo menstrual.

**Resultados:**

Los estudios revisados evidencian que el estigma menstrual tiene un impacto multidimensional que se manifiesta en la exclusión social, la medicalización del cuerpo menstruante, la desatención institucional y la persistencia de prácticas discriminatorias. La pobreza menstrual, identificada en contextos de ingresos bajos y también en países de renta alta, repercute en la salud física, emocional y social de las personas menstruantes. Asimismo, se destaca la insuficiencia de políticas públicas inclusivas, la escasa representación en los marcos normativos laborales y la reproducción de discursos estigmatizantes en los medios. Sin embargo, se identifican también avances en el activismo, en las estrategias educativas y en iniciativas legislativas emergentes.

**Conclusiones:**

Los resultados subrayan la necesidad de promover políticas públicas interseccionales que aborden la salud menstrual desde una perspectiva de derechos humanos, equidad de género y justicia social. Asimismo, se propone reforzar la alfabetización menstrual, mejorar el acceso a productos e infraestructuras adecuadas, e integrar el enfoque de salud menstrual en los sistemas educativos, sanitarios y laborales, como vía para garantizar una vivencia menstrual digna, saludable y equitativa.

## Introduction

The menstrual cycle comprises a series of physiological and endocrine processes characterized by changes in the synthesis, release, transport, and action of female sex hormones in target organs [1]. These mechanisms facilitate ovulation and enable fertilization, an essential process for reproduction. In the absence of fertilization, the endometrium sheds, giving rise to menstruation and marking the beginning of a new menstrual cycle [1]. As part of the life course, menstruation is a recurring biological process involving the shedding of the inner lining of the uterus [2] and influencing both the physical and psychological well-being of menstruating people. The menstrual cycle spans a significant portion of life, beginning in adolescence with menarche and ending around age 50 with menopause [3], defined as the final menstrual period and the cessation of ovulation, signalling the end of reproductive life [4].

Despite its natural character, menstruation has historically been subject to numerous taboos, myths, and beliefs associating it with impurity, weakness, or danger, leading to a deeply rooted stigma [5, 6]. These sociocultural constructions have rendered menstrual experience invisible, confining it to the private and shameful sphere and limiting access to information, resources, and safe spaces for menstrual management [7]. According to the classical definition of stigma [8], this concept refers to a deeply discrediting attribute that marks individuals as inferior or threatening. In this sense, the set of negative attitudes, beliefs, and practices surrounding menstruation has contributed to its social stigmatization [9].

Menstrual stigma persists globally [10], sustained by silence [11] and by narratives linking menstruation with impurity and shame [6], often portraying it as an intimate, hidden, or even mysterious matter [12]. This concealment has generated widespread misinformation and a lack of understanding of this physiological process, creating substantial obstacles to managing menstruation in public spaces [7]. Historically, menstruation has been approached within biological and medicalized frameworks [13], which depict menstruating bodies as problematic and pathologize a natural process [14]. In turn, labour legislation has not incorporated recognition of the needs associated with menstruation, reinforcing the notion that the menstruating body is defective or insufficient [13, 14].

In this context, the concept of menstrual justice has emerged as a comprehensive framework advocating for a dignified, healthy, and discrimination-free menstrual experience [15]. Legal and sociopolitical scholarship has deepened this notion, defining menstrual justice as a framework grounded in human rights, gender equity, and social justice principles aimed at dismantling the structural inequalities and stigmas that restrict autonomy, well-being, privacy, and social participation [16]. From a public health perspective, menstrual inequalities hinder equitable access to resources, information, and services, contributing to broader gender inequities within health systems [17]. Menstrual justice therefore encompasses not only access to menstrual products, infrastructures, and evidence-based education, but also the transformation of sociocultural norms and institutional structures that perpetuate inequality [15–18].

Gender equity is fundamental to these debates and refers to the fair and context-sensitive distribution of opportunities, resources, and responsibilities among people of different genders, recognising the historical and structural inequalities that affect access to health, education, employment, and social participation. In the field of reproductive health, it entails addressing the barriers menstruating people face in accessing safe menstrual management and reproductive health services across the life course. Additionally, international frameworks such as the Sustainable Development Goals—specifically SDG 3 (Good Health and Well-being) and SDG 5 (Gender Equality)—explicitly situate menstrual health within global commitments to human rights, public health, and gender justice [2].

Nonetheless, despite growing political and social interest in menstruation, significant gaps persist in the scientific literature regarding the psychosocial impacts of stigma and discrimination in different contexts. Few studies have systematically examined how menstrual stigma manifests, how it affects well-being, and how it shapes social participation, or analysed the barriers to implementing public policies aimed at promoting menstrual equity [5, 6, 9]. Likewise, academic production from Spanish-speaking contexts—particularly Latin America and Spain, where menstrual activism, community-based strategies, and policy innovations are especially prominent—has been incorporated only to a limited extent into international syntheses, reducing the visibility of contextualised conceptual contributions and lived experiences [7].

In this context, the present study aims to analyse the current state of knowledge on menstrual stigma and discrimination from a psychosocial perspective, examining recent contributions relevant to educators, healthcare professionals, policymakers, and researchers working to advance menstrual equity and justice [2]. In particular, it emphasises the importance of transforming cultural perspectives, increasing visibility, and promoting adequate regulatory frameworks, highlighting the central role of communication as an organising axis in the construction of shared cultural values [4].

Within this framework, the study addresses the following research question: How do stigma and discrimination shape menstrual experiences and contribute to gender inequities in access to reproductive healthcare? To answer this question, this review analyses the forms of menstrual stigma experienced by menstruating people and the different manifestations of discrimination they encounter, with the aim of deepening understanding of how sociocultural factors perpetuate health- and gender-related inequalities.

## Methods

### Search strategy

This study employed a narrative literature review as its methodological approach. As a non-original research article, this format allowed for the selection, compilation, and analysis of existing scientific literature on menstrual stigma and discrimination across different social and cultural contexts. This type of review is particularly well suited to the subject matter, given its flexibility to explore in depth the complexity of menstrual stigma and its intersections with social, cultural, historical, educational, and health-related factors. It also enables the integration of diverse perspectives, theoretical frameworks, methodologies, and analytical approaches, while supporting the identification of future research needs and potential intervention pathways.

The review was conducted between 2019 and 2025, using the databases Dialnet, PubMed, and BioMed Central. These platforms were selected due to the complementary nature of their coverage. Dialnet offers multidisciplinary access with a strong focus on humanities and social sciences, particularly in Spanish-language publications from Ibero-American contexts. PubMed specialises in the health sciences and includes a broad range of peer-reviewed, predominantly English-language publications of international scope. BioMed Central provides extensive access to open-access journals in public and global health, contributing to transparency and visibility in scientific research.

The search strategy combined keywords in both Spanish and English—“Estigma”, “Menstruación”, “Discriminación”, “Ciclo menstrual”, “Stigma”, “Menstruation”, “Discrimination”, and “Menstrual cycle”—using advanced search tools and Boolean operators (“AND”, “OR”) in different combinations. Filters for publication date and full-text availability were applied. Only articles written in Spanish or English were considered.

### Eligibility criteria

#### Inclusion criteria

Studies were included if they met all of the following criteria: (a) published within the last seven years to ensure the incorporation of recent and relevant evidence; (b) available in open-access format to allow full consultation of their content; (c) written in Spanish or English; and (d) explicitly addressing topics related to menstruation, stigma, and discrimination, situated in social, healthcare, or educational contexts.

#### Exclusion criteria

Articles that did not meet these conditions were excluded. Specifically, studies were excluded if they: (a) were not available in full-text format; (b) were published outside the defined time frame; (c) were written in languages other than Spanish or English; (d) consisted of non-scientific materials such as opinion pieces, non-peer-reviewed reports, or media publications; or (e) did not directly or analytically engage with the core topics of menstruation, stigma, and discrimination.

## Results

The literature search yielded a total of 905 records. After applying the predefined inclusion and exclusion criteria, 36 studies were selected for analysis. These studies encompassed a variety of methodological designs, including reviews, descriptive studies, original research, comparative studies, qualitative studies, theoretical analyses, cross-sectional and psychometric studies, as well as one longitudinal and two empirical studies.

### Menstrual stigma

Menstruation is a natural biological process involving the release of blood from the uterus [19], accompanied by cognitive, physical, emotional, and social changes [20]. Before bleeding begins, premenstrual symptoms—such as irritability or breast pain—may occur and typically subside once menstruation starts. During the first days of the cycle, abdominal and back pain, breast tenderness, and dysmenorrhea are common [21, 22]. These physical discomforts may amplify emotional distress, irritability, depressive feelings, or lowered self-esteem [22]. Some studies report that, for tens of millions of women worldwide, menstruation disrupts physical, mental, and social well-being [23]. In line with this, research conducted in Sweden and Japan shows that menstrual symptoms limit daily functioning, social relationships, and sexual and mental health, negatively affecting health-related quality of life [24, 25].

Experiences of menarche and menstruation are shaped by sociocultural beliefs and practices, which influence perceptions of menstruation and contribute to its stigmatization [6, 26, 27]. Considering menstruation as a private or hidden matter restricts open dialogue and knowledge-sharing, perpetuating misinformation and inadequate menstrual education [12, 28]. In this way, menstrual stigma negatively affects personal, social, and economic well-being and is commonly associated with shame, discomfort, and secrecy, which can harm physical and mental health and overall quality of life [11, 29]. Beyond secrecy, stigma can hinder accurate diagnosis and treatment of menstrual health problems by discouraging communication with healthcare professionals [24, 30].

Across several low- and middle-income countries (LMICs) in sub-Saharan Africa, inadequate options for disposing of menstrual products intensify feelings of shame and pose health risks [31]. In Ghana, menstruation is associated with multiple difficulties for adolescent girls [32]. In humanitarian and displacement settings, public spaces are perceived as unsafe, marked by fear and social isolation due to stigma [31], while limited access to accurate sexual and reproductive health information fosters misconceptions. In Ethiopia, participants reported feelings of shame, dehumanization, and isolation during menstruation [26]. Significant associations have also been found between cultural beliefs and women’s mental health [27]. The secrecy surrounding menstruation restricts open conversations and menstrual education, particularly in low-resource settings [31], where normalizing open dialogue may foster supportive environments and enhance girls’ self-esteem and comfort during their periods [26].

Similar patterns appear across several Asian countries. A study conducted in Syria found that menstrual matters are expected to remain hidden, making discussion of menstruation, menstrual products, pain, or symptoms socially unacceptable [33]. In India, concerns about privacy, stigma, and limited access to services discourage adolescents from seeking sexual and reproductive health information or care [34]. In South Korea, menstruation is described as a “secret area” reserved for women, which hinders men’s ability to understand or show interest [27].

In Europe, a qualitative study conducted in the Netherlands found that menstrual information was perceived as insufficient and that a knowledge gap between men and women persists, partly due to male disinterest. This lack of awareness contributes to difficulties in social and workplace contexts due to limited empathy and understanding [28].

Taken together, findings from diverse cultural settings reveal that menstrual blood is frequently considered impure or dangerous, imbued with symbolic meanings that influence disposal practices. These beliefs are reinforced by stigma and secrecy [32] and are associated with restrictive practices and taboos [27]. As a result, menstruating bodies are often portrayed as “fragile” or “imperfect,” intensifying stigma. Menstruation itself is frequently described as “disgusting” or “contaminating” [12], and certain religious teachings have perpetuated the notion that menstrual blood is more impure than other bodily fluids [6]. Some studies suggest that menstrual blood functions as a stigmatizing marker that reinforces sexual taboos, including the avoidance of intimacy during menstruation [9].

From a critical perspective, some cultural expressions have reinforced biomedical discourses associating menstruation with biologicism and the psychologization of women [35]. Biologicism frames menstruation solely as a biological phenomenon, disregarding psychological and sociocultural dimensions. Psychologization, on the other hand, assumes that women’s problems stem primarily from emotions rather than organic causes. Both perspectives overlook the social and cultural components of menstruation and reinforce reproductive-centered narratives, contributing to ignorance and pathologization [35].

In recent years, menstrual health has increasingly been recognized as a human rights and public health issue and incorporated into the 2030 Agenda for Sustainable Development [36]. The Global Menstrual Collective’s Terminology Action Group [37] defines menstrual health as a state of physical, mental, and social well-being—and not merely the absence of disease—in relation to the menstrual cycle [38]. This definition aligns with the World Health Organization’s conceptualization of health and underscores the importance of mental and social well-being, elevating the understanding of menstrual stigma and its consequences as structural health and equity challenges that must be addressed.

### Structural discrimination

Menstrual stigma has a particularly harmful impact on girls’ education, leisure opportunities, physical activity, and processes of self-isolation, leading to the exclusion of women and other menstruating people from cultural, social, and religious activities [31]. Likewise, the difficulty of speaking openly about menstruation with friends, family members, or healthcare professionals [22] generates uncertainty about which symptoms are normal and which may require medical attention. The fear of embarrassment—especially due to potential menstrual accidents or the absence of adequate products—causes many girls to miss school during their periods [25]. In addition, socioeconomic vulnerability limits access to essential products and services, shaping menstrual inequalities along lines of gender, class, and race [31].

Similar patterns are observed across several Asian countries. In India, adolescents face substantial barriers to accessing both menstrual management products and sexual and reproductive health information, limiting their knowledge about menstruation [29]. In Sub-Saharan Africa, many girls miss school due to menstrual pain, lack of pads, and fear of leakage, while cultural restrictions further constrain their participation in daily activities [25]. At the same time, women in Nigeria describe how menstruation reinforces their exclusion from everyday life [25]. More broadly, the absence of comprehensive education on menstruation and reproductive health [39], combined with sociocultural factors, often leads to inadequate health practices [14].

In the European context, recent studies indicate that menstrual literacy remains insufficient. In Spain, although health-related content has gradually been incorporated into the educational system, women’s health issues are still not addressed explicitly [34]. As a result, menstrual knowledge comes from both formal (school) and informal (family and peers) sources, favouring the transmission of myths and misinformation [24]. Overall, adequate menstrual literacy is essential for supporting informed health-related decision-making [22].

Globally, low levels of menstrual literacy are also documented, a trend widely reported in current research [22, 24, 28]. This lack of knowledge—exacerbated by stigma and taboos—reinforces gender inequalities and produces social consequences such as school and workplace absenteeism, as well as reductions in quality of life [28]. Moreover, other studies highlight that these challenges are compounded by the underrepresentation of women and women’s health issues in scientific research, limiting the development of informed and context-appropriate responses [23].

### Menstrual poverty

Menstrual poverty is defined as the lack of access to affordable menstrual products, adequate information, and safe conditions for menstrual management—dimensions increasingly recognised as key public health and equity concerns [2]. Moreover, several studies indicate that limited access to affordable products often leads individuals to rely on improvised or unhygienic alternatives, which increases the risk of infection and contributes to feelings of shame and discomfort [6, 27, 37]. In this way, inadequate management of menstrual pain and self-medication emerge as direct consequences of stigma and the lack of appropriate resources [7].

In the Global South, socioeconomic vulnerability has been identified as a major determinant of menstrual poverty. The commodification of menstrual products—treated as market goods—perpetuates inequalities and restricts access to safe options [31]. Similarly, in sub-Saharan Africa, multiple studies show that limited access to affordable menstrual products increases infection risk and generates feelings of shame that negatively affect self-esteem [16, 18]. In Ghana, for instance, the high cost and limited availability of products continue to pose barriers, with implications for reproductive health, social isolation, and girls’ academic performance [27]. Among displaced or refugee populations, these challenges are further exacerbated by the lack of clean water, adequate sanitation, and menstrual-specific hygiene infrastructures [25, 37].

Across Asia, studies from Nepal highlight persistent menstrual-related restrictions and superstitions, although women’s perspectives on these practices remain insufficiently documented [35]. Likewise, in India, menstrual health management continues to be strongly shaped by sociocultural norms that restrict mobility and access to basic services [36]. In Oceania, both urban and rural areas of Pacific Island countries and territories face recurring obstacles due to insufficient WASH (water, sanitation, and hygiene) facilities in schools, workplaces, and broader community settings [21].

Menstrual poverty also affects high-income countries. In these contexts, factors such as the gender pay gap, labour precarity, and the high cost of hygiene products may hinder regular access to menstrual products [20]. In Spain, menstrual inequities are particularly prevalent among people experiencing socioeconomic vulnerability, migrant communities, and trans or non-binary menstruators [15]. Additionally, people experiencing homelessness face substantial barriers—including limited access to products, safe spaces, and specialised services—which hinder the dignified management of menstruation [37].

Taken together, the literature indicates that menstrual poverty has negative effects on physical and emotional health, represents a significant barrier to gender equality, and undermines human rights and social justice [15]. Consequently, governments play a central role in reducing these disparities by implementing legislative and policy measures that ensure the economic and material accessibility of menstrual products [2]. In parallel, there is growing recognition that menstrual health is essential not only for gender equality but also for the overall well-being of all individuals [37].

### Media representations

Digital media and social networks now play a prominent role in disseminating information related to menstruation [6]. Recent analyses indicate an increase in public discussions about menstruation across online platforms, social media, and contemporary literature, contributing to shifts in perceptions and beliefs about the menstrual cycle [6]. Additionally, ecofeminist and community-based movements have fostered digital environments that promote conscious and positive approaches to menstruation, enabling menstruating individuals to share information, experiences, and perspectives. These online networks exert a particularly strong influence among younger generations.

In parallel, the widespread adoption of mobile health technologies has facilitated advances in menstrual health literacy, communication, and education. Mobile applications offering cycle tracking, symptom monitoring, and menstrual health information are increasingly used as tools to support knowledge and self-management [26]. Social media influencers also appear to contribute to the normalization of menstruation and the reduction of menstrual stigma, according to qualitative accounts from menstruating individuals [38]. While many people receive their initial information about menstruation through family, school, or peers, digital media often becomes the primary source of information in adulthood [15]. Consequently, online media and social networks represent a promising public health strategy for improving menstrual literacy. Nonetheless, important challenges remain, including the spread of misinformation and the reinforcement of inequalities associated with the digital divide [15].

At the same time, technological developments and media influence have accelerated the circulation of medicalised discourses surrounding menstruation [14]. Advertising for menstrual hygiene products—such as pads and tampons—often reinforces norms of concealment by promoting the idea that menstruation should remain invisible. These advertisements typically emphasise the capacity of products to hide fluids and odours, remain discreet under clothing, and minimise any visible evidence of menstruation [13]. The use of terms such as “leaks” isolates menstrual blood as something undesirable and reinforces social expectations that menstruation should not be seen, contributing to a wider culture of shame. This culture teaches menstruating individuals that staining in public constitutes a deeply embarrassing event.

Furthermore, many media messages—particularly in the United States—portray menstruation as an undesirable phenomenon and collectively reinforce stereotypes about menstruating people [13]. Such representations frequently describe menstruation as debilitating, problematic, or inherently negative. These narratives strengthen menstrual stigma and shape social attitudes across diverse demographic groups [13].

### Labour legislation

In the labour sphere, economic and organisational constraints can hinder the adoption of menstrual health policies, as some employers may resist change due to perceived financial costs or concerns about productivity [10]. Within this context, regulating employment conditions for women and menstruating individuals is essential to prevent gender-based discrimination. However, in many countries, existing protective measures continue to focus primarily on maternity, leaving menstruation largely absent from labour legislation and social policy frameworks [40].

Moreover, international human rights bodies have emphasised that menstruation should be recognised as a matter of health and human rights rather than merely an issue of personal hygiene [40]. The concept of menstrual health, which has gained increasing visibility in global agendas related to health, education, human rights, and gender equality, has been strengthened by the efforts of community-based activists and healthcare professionals. As a result, a growing number of international organisations—including the World Health Organization—now explicitly identify menstrual health as a policy priority. Nevertheless, despite having multiple conventions and recommendations addressing gender discrimination and maternity protection, the International Labour Organization (ILO) does not include specific references to menstruation in workplace regulations [40].

Across the European context, this regulatory gap is particularly evident. For instance, Spanish legislation mandates general requirements concerning workplace sanitary facilities but does not address the provision of menstrual hygiene products or explicit guidance for adequate menstrual management. Similarly, Swedish labour environment law contains no specific mention of menstruation, and in Hungary, although occupational safety regulations include provisions for women, they do not explicitly regulate menstrual waste disposal [40]. More broadly, the comprehensive report by the European Agency for Safety and Health at Work, New Risks and Trends in the Occupational Safety and Health of Women at Work, includes the word “menstruation” only once within its 382 pages—and solely in reference to reproductive health [41]. This scant presence illustrates the extent to which menstruation has not been systematically integrated into labour regulation or occupational risk-prevention policies.

At the same time, evidence indicates that menstrual symptoms do not typically lead to absenteeism but rather to presenteeism—the practice of attending work while experiencing pain or discomfort due to fear of negative labour consequences [40]. In response, some countries have introduced menstrual leave policies that allow workers to take time off due to menstrual-related difficulties. However, such measures remain limited and are primarily concentrated outside Europe, in countries such as Japan, Indonesia, South Korea, Zambia, several Chinese provinces, Mexico, Taiwan, the United Arab Emirates, and one province in Argentina. Recently, Spain became the first EU Member State to legally implement paid menstrual leave, marking a significant step forward in public policy. Nonetheless, various organisations have expressed concerns that this measure may inadvertently reinforce employer bias or reduce women’s competitiveness in the labour market [40].

Despite these challenges, an increasing number of companies worldwide have begun incorporating menstrual health considerations into workplace policies. These initiatives include flexible working arrangements, menstrual leave, provision of menstrual products, and other supportive measures. Collectively, they point toward growing recognition of the need to integrate menstrual health into workplace environments and the broader rights of workers.

### Menstrual activism

Menstrual activism has played a significant role in challenging the stigma and silence surrounding menstruation and in advancing more equitable, rights-based approaches to menstrual health [6]. Various movements have contributed to reframing menstruation as a natural bodily process, emphasizing that the negativity associated with it is socially and culturally constructed rather than inherent [13]. In this regard, menstrual activism seeks to empower menstruating individuals by encouraging open dialogue, promoting accurate information, and contesting cultural narratives that portray menstruation as shameful or problematic [9].

From a broader sociocultural perspective, activist initiatives also highlight the importance of fostering positive and autonomous meanings of menstruation, resisting discourses that depict it as uncomfortable, dirty, or burdensome [42]. These approaches advocate for practices grounded in rest, bodily awareness, self-care, and resistance to productivity-driven norms that often fail to acknowledge menstrual needs. Similarly, activism around menstrual products promotes alternatives such as reusable pads and menstrual cups, which are framed as more sustainable and affordable options and as tools for enhancing autonomy and reducing waste [42].

At the global level, international organizations—including the United Nations and various NGOs—have called for reducing gender gaps in education and promoting dignified menstrual management through improvements in water, sanitation, and hygiene (WASH) infrastructure, as well as increased access to menstrual products and comprehensive reproductive health education [43]. Despite these efforts, substantial inequalities persist. Many low- and middle-income countries continue to face barriers in menstrual health management, where adolescents may rely on inadequate hygiene practices due to limited knowledge, cultural restrictions, or infrastructural deficits [16].

Against this backdrop, several intervention initiatives have demonstrated promising results. In sub-Saharan Africa, programs that integrate menstrual health education, community dialogue, and product distribution have contributed to reducing stigma, strengthening girls’ self-esteem, and improving menstrual management conditions [25]. In refugee settings in Uganda, Somaliland, Madagascar, Tanzania, and Burundi, multifaceted interventions—ranging from the distribution of disposable and reusable menstrual products to training for healthcare personnel—have enhanced access to menstrual resources and improved the integration of menstrual health within reproductive health services [25]. Ensuring access to adequate water and sanitation facilities remains essential, as hygienic menstrual management is virtually impossible without proper infrastructure [25].

In Asia, digital education initiatives in India have improved menstrual health knowledge and familiarity with hygiene products among adolescents through mobile-based, interactive interventions [29]. In Nepal, a range of awareness and promotion strategies has been implemented over the past decade to challenge menstrual taboos and support girls’ participation in school, work, and daily life [44]. In South Korea, recent studies note a shift toward more student-centered pedagogical models in sexual and menstrual health education [23].

In high-income countries, “menstrual policies”—such as the provision of free menstrual products in schools, workplaces, and community settings—have gained visibility and support [45]. In Europe, pioneering initiatives in Sweden and Norway have expanded access to free menstrual products, while fiscal strategies aimed at reducing or eliminating taxes on menstrual products have contributed to greater affordability [18]. Spain, for instance, has reduced value-added tax (VAT) on menstrual products from 10% to 4%, recognizing them as essential goods [15]. In countries such as the United Kingdom, Kenya, India, Canada, Australia, and Germany, these taxes have been completely eliminated [45]. Finally, recent analyses highlight shifting social attitudes, marked by a decline in restrictive discourses and increasing efforts to normalize menstruation through comprehensive health education policies [36].

## Discussion

The findings of this review show that menstrual stigma operates as a structural phenomenon that transcends geographical and economic contexts. Rather than a collection of isolated cultural practices, the studies analysed illustrate that the discourses surrounding menstruation are sustained by gendered logics, material inequalities, and biomedical assumptions that recur—albeit with varying intensity—across all examined regions. This pattern aligns with previous literature indicating that menstrual stigma is not solely rooted in traditional taboos but also in inadequate infrastructures, incomplete educational frameworks, and gaps in labour regulation [26, 31].

Additionally, the results situate this review within a broader body of work conceptualizing menstruation as a symbolic field shaped by cultural values, social inequalities, and power relations [6, 21]. This perspective helps explain why, even in high-income settings, negative or medicalized representations of the menstrual cycle persist—a phenomenon that has been linked to the historical tendency to frame the female body as deficient, fragile, or problematic [30]. In this way, the findings confirm that menstrual stigma cannot be explained exclusively through traditional practices; rather, it forms part of a wider network of social norms and medical epistemologies that influence how menstruation is understood, discussed, and managed.

In parallel, the recurrent presence of inequalities in access to menstrual products, information, and WASH infrastructures suggests that menstruation is also a matter of distributive justice. This approach broadens the analytical lens and allows the results to be connected to the emerging international debate on menstrual justice, which integrates human rights, gender equity, and the structural determinants of health [2]. Unlike studies focused solely on taboo, menstrual justice places emphasis on the material and institutional conditions that generate and sustain vulnerability. From this perspective, the findings of this review reinforce the argument that menstrual poverty is not simply an economic consequence but an indicator of systemic inequalities closely tied to autonomy and bodily dignity.

Another notable contribution concerns the growing role of digital media in shaping perceptions and narratives about menstruation. While some studies show that social media platforms can disseminate stigmatizing messages, others highlight their potential to serve as spaces for resistance, peer education, and the circulation of alternative discourses—particularly among younger generations [15, 38]. This dual role echoes other research in digital health, underscoring the need to approach menstrual communication not merely as an individual attitudinal issue but as a matter of informational governance.

Finally, this review reveals that menstruation remains largely absent from legal and policy frameworks, including in countries where some progress has been made. This regulatory gap aligns with what other authors describe as the institutional invisibility of the menstrual cycle, which prevents menstruation from being recognized as a relevant factor in occupational health, well-being, and equity [40, 41]. Taken together, the findings suggest that the cultural transformation called for by menstrual justice must be accompanied by more robust institutional and regulatory development.

### Limitations

This review has several limitations. First, only open-access studies were included, which may restrict the representativeness of the analysed literature. Second, the methodological heterogeneity of the studies complicates direct comparison and limits the identification of universal patterns. Third, there is a widespread lack of longitudinal research on menstrual experiences—an evidence gap repeatedly highlighted in the literature [26]. Finally, menstrual experiences are deeply shaped by cultural, social, and economic factors, which limits the transferability of the findings.

## Conclusions

The findings of this review demonstrate that menstrual stigma is a complex phenomenon with cultural, economic, and institutional roots that transcend national borders. They also confirm that menstruation constitutes a site where structural inequalities and discriminatory mechanisms converge, affecting rights, opportunities, and overall well-being. For this reason, menstrual justice emerges as a relevant conceptual framework for guiding future policies, programmes, and research. At the same time, the persistence of gaps in legislation, education, and workplace environments highlights the need for comprehensive strategies that combine cultural transformation, material redistribution, and institutional recognition.

Nevertheless, this review reinforces the importance of continuing to develop actions that promote the health, dignity, and equity of menstruating individuals across all social domains.

## Data Availability

All data used in this narrative review are drawn from published studies and are publicly available in their respective sources. No new datasets were generated for this study, or Not applicable.
